# Supplementary material for: Implications of Poly(A) Tail Processing in Repeat Expansion Diseases
Source: Cells. 2022 Feb 15;11(4):677. doi: 10.3390/cells11040677 (PMC8870147; doi:10.3390/cells11040677)
Supplement: Supplementary file 1 [file cells-11-00677-s001.zip › cells-1580366-supplementary.pdf]

Table S1. Repeat expansion diseases.

	Disease	Mutant gene	Repeat motif	Repeat tract location
1	**Benign adult familial myoclonic epilepsy (BAFME)**	*SAMD12*	e.g. TTTCA	Intron
2	**Baratela-Scott Syndrome**	*XYLT1*	GGC	Promoter
3	**C9ORF72-mediated ALS/FTD**	*C9ORF72*	GGGGCC	Intron
4	**Cerebellar ataxia, neuropathy, and vestibular areflexia syndrome (CANVAS)**	*RFC1*	AAGGG	Intron
5	**Cleidocranial dysplasia (CCD)**	*RUNX2*	GCN	Exon
6	**Congenital central hypoventilation syndrome (CCHS)**	*PHOX2B*	GCN	Exon
7	**Myotonic dystrophy type 1 (DM1)**	*DMPK*	CTG	3'-UTR
8	**Myotonic dystrophy type 2 (DM2)**	*CNBP1*	CCTG	Intron
9	**Dentatorubro-pallidoluysian atrophy (DRPLA)**	*ATN1*	CAG	Exon
10	**Familial adult myoclonic epilepsy types 1-7 (FAME 1-7)**	Various	TTTCA	Intron
11	**Fuchs endothelial corneal dystrophy (FECD)**	*TCF4*	CTG	Intron
12	**Fragile XE syndrome (FRAXE)**	*AFF2/FMR2*	CCG	5'-UTR
13	**Friedreich ataxia (FRDA)**	*FXN*	GAA	Intron
14	**Fragile X syndrome; Fragile X-associated tremor/ataxia syndrome (FXS; FXTAS)**	*FMR1*	CGG	5'-UTR
15	**Glutaminase deficiency**	*GLS*	CAG	5' UTR
16	**Huntington's disease (HD)**	*HTT*	CAG	Exon
17	**Huntington disease-like 2 (HDL2)**	*JPH3*	CTG	Exon
18	**Hand–foot–genital syndrome (HFGS)**	*HOXA13*	GCN	Exon
19	**Holoprosencephaly 5 (HPE5)**	*ZIC2*	GCN	Exon
20	**Jacobsen syndrome**	*CBL2*	CCG	5'-UTR
21	**Neuronal intranuclear inclusion disease (NIID)**	*NOTCH2/NLC*	CGG	5'-UTR

**22**	**Oculopharyngeal muscular dystrophy (OPMD)**	*PAPBN1*	GCG	Exon
**23**	**X-linked hypopituitarism (PHPX)**	*SOX3*	GCN	Exon
**24**	**Spinal and bulbar muscular atrophy (SBMA)**	*AR*	CAG	Exon
**25**	**Spinocerebellar ataxia type 1 (SCA1)**	*ATXN1*	CAG	Exon
**26**	**Spinocerebellar ataxia type 2 (SCA2)**	*ATXN2*	CAG	Exon
**27**	**Spinocerebellar ataxia type 3 (SCA3)**	*ATXN3*	CAG	Exon
**28**	**Spinocerebellar ataxia type 6 (SCA6)**	*CACNA1A*	CAG	Exon
**29**	**Spinocerebellar ataxia type 7 (SCA7)**	*ATXN7*	CAG	Exon
**30**	**Spinocerebellar ataxia type 8 (SCA8)**	*ATXN8/ATXN8OS*	CAG/CTG	3’-UTR
**31**	**Spinocerebellar ataxia type 10 (SCA10)**	*ATXN10*	ATTCT	Intron
**32**	**Spinocerebellar ataxia type 12 (SCA12)**	*PPP2R2B*	CAG	5’-UTR
**33**	**Spinocerebellar ataxia type 17 (SCA17)**	*TBP*	CAG	Exon
**34**	**Spinocerebellar ataxia type 31 (SCA31)**	*TK2* and *BEAN*	TGGA	Intron
**35**	**Spinocerebellar ataxia type 36 (SCA36)**	*NOP56*	GGCCTG	Intron
**36**	**Spinocerebellar ataxia type 37 (SCA37)**	*DAB1*	ATTTC	Intron
**37**	**Unverricht–Lundborg disease (ULD)**	*CSTB*	CCCCGCCCCGCG	Promoter
**38**	**X-linked dystonia parkinsonism (XDP)**	*TAF1*	CCCTCT	Intron
**39**	**X-linked intellectual disability (XLMR)**	*ARX*	GCG	Exon
